# Anatomic and operative predictors of aortic expansion following aortic dissection repair

**DOI:** 10.1038/s41598-025-11286-2

**Published:** 2025-07-11

**Authors:** Ryaan EL-Andari, Sabin Bozso, Yongzhe Hong, Michael Moon

**Affiliations:** 1 Division of Cardiac Surgery, University of Alberta, Edmonton, AB Canada; 2 Mazankowski Alberta Heart Institute, University of Alberta, 11220 83 Ave NW, Edmonton, AB Canada

**Keywords:** Aortic dissection, Aortic remodeling, Aortic surgery, Aortic diseases, Outcomes research

## Abstract

Following surgical repair of acute type A aortic dissection(ATAAD), distal aortic degeneration and growth may occur. Previous evidence has suggested that false lumen(FL) communications and flow may influence postoperative aortic remodeling, although the contribution of branch vessel dissection and FL communications is unclear. Patients who underwent ATAAD repair from 2017 to 2023 at a single center with at least 1 year of follow-up imaging were included in this study. Patients were grouped based on aortic pathology and surgical repair. Preoperative and postoperative measurements were taken at the level of zone 1 and between zones 4/5. 63 patients were included in this study. 87.3% received a hemiarch repair, 34.9% received hemiarch + AMDS Hybrid prosthesis, and 12.7% received a total arch replacement. Proximal aortic remodeling was not reliably predicted by the presence of FL communications or surgical approach. Distal aortic growth was independently associated with ≥ 3 or 4 visceral vessel dissections(*p* = 0.04 − 0.005). In conclusion, distal aortic remodeling following ATAAD repair was predicted by visceral vessel involvement. While the aortic arch is often emphasized at the time of ATAAD repair, features of the distal aorta may help to risk stratify patients for long-term adverse events, helping to guide initial management and post-ATAAD repair follow-up.

**Supplementary Information:**

The online version contains supplementary material available at 10.1038/s41598-025-11286-2.

## Introduction

Acute type A aortic dissection(ATAAD) is a surgical emergency requiring urgent intervention to reduce the risk of death or significant morbidity such as stroke, visceral organ ischemia, and hemorrhage, among others^1^. The goal of ATAAD repair is to resect the primary entry tear, stabilize the aortic arch, prevent progression of dissection, and address organ malperfusion^1–4^. A hemiarch replacement is often the minimum intervention necessary intervention to prevent mortality and stabilize the aortic arch. The decision for the extent of the distal repair depends largely on anatomical features and surgeon experience. Aortic arch tears, connective tissue disorders, patient age, and visceral organ malperfusion may influence the decision towards a more extensive arch intervention. Aortic arch interventions are often pursued with the goal of resecting entry tears, treating malperfusion, and promoting positive long-term remodeling^2,3,5–8^. Regardless of surgical approach, distal aortic expansion may still occur. A driving force for aortic degeneration following ATAAD repair and required reoperation is false lumen(FL) growth as a result of FL pressurization^4,9,10^. In cases of hemiarch repair, dissected head vessels and true lumen(TL) to FL communications may continue to pressurize the FL resulting in distal aortic dilation, and while this may also occur with involved branch vessels, this relationship has not been conclusively proven. In patients who have undergone total arch replacement (TAR), these communications in the head vessels are excluded and cannot contribute to distal aortic remodeling. Therefore, we sought to elucidate the contribution of head vessel and visceral vessel communications with the remaining FL to distal aortic expansion in patients who have undergone ATAAD repair.

## Results

### Baseline demographics

123 patients underwent repair of type A dissection during the study period. 60 were excluded if the aortic dissection did not involve the head vessels, did not have surgical repair, or did not have a 1 year postoperative CT scan. 63 consecutive patients that met the inclusion criteria were included in this study(Table 1). The average age was 60 years old. Males comprised 70.6% (*n* = 46) of the patient population. Common comorbidities included hypertension (*n* = 45, 66.2%), dyslipidemia (*n* = 12, 17.6%), smoking (*n* = 10, 15.8%), atrial fibrillation (*n* = 5, 7.4%), and cerebrovascular disease (*n* = 5, 7.4%). On presentation, 6.3% (*n* = 4) of patients had aortic rupture and 7.4% (*n* = 5) had tamponade. 20.6% of patients (*n* = 13) presented with renal malperfusion, 14.3% (*n* = 9) with extremity malperfusion, 7.4% (*n* = 5) with cerebral malperfusion, and 4.4% (*n* = 3) with mesenteric malperfusion(Table 1).

**Table 1:** Baseline demographics and operative characteristics of the included study population.

Baseline Demographics	*N* = 63 (%)
Age (Mean)	60
Male Sex	46 (70.6)
Hypertension	45 (66.2)
Dyslipidemia	12 (17.6)
Smoking	10 (15.8)
Atrial Fibrillation	5 (7.4)
Cerebrovascular Disease	5 (7.4)
CHF	3 (4.4)
Coronary Artery Disease	2 (2.9)
COPD	2 (2.9)
Diabetes	2 (2.9)
Tamponade	5 (7.4)
Aortic Rupture	4 (6.3)
Malperfusion	
- Renal	13 (20.6)
- Extremity	9 (14.3)
- Cerebral	5 (7.4)
- Mesenteric	3 (4.4)
Peripheral Vascular Disease	2 (2.9)
BMI (Mean)	29 kg/m²
**Operative Characteristics**	***N*** **= 63 (%)**
Aortic Valve Replacement	4 (6.3)
Aortic Valve Repair	14 (22.2)
Bentall Procedure	23 (36.5)
Ascending Aortic Replacement	63 (100)
Hemiarch	55 (87.3)
AMDS Hybrid Prosthesis	22 (34.9)
Total Arch	8 (12.7)
Coronary Artery Bypass Grafting	5 (7.9)
Mitral Valve Procedure	2 (3.2)

COPD, chronic obstructive pulmonary disease; CHF, congestive heart failure; BMI, body mass index.

Patient groups included the following. Total proximal cohort *n* = 55 (excluding TAR), distal entire cohort *n* = 63 (including all patients). In the group with proximal measures, 33 underwent a hemiarch repair while 22 underwent an extended arch repair. 13 patients had SAVD and a hemiarch repair, 20 had FL communications and a hemiarch repair, 9 had SAVD and an AMDS repair, and 13 had FL communications and an AMDS repair.

In the group with distal measures, 33 patients underwent a hemiarch repair and 30 underwent an extended arch repair. 13 patients had SAVD and a hemiarch repair, 20 had FL communications and a hemiarch repair, 9 had SAVD and an AMDS repair, 13 had FL communications and an AMDS repair, and 8 had a total arch repair.

### Operative characteristics

Among the included patients, 100% (*n* = 63) received an ascending aortic replacement. Aortic valve replacement was required in 6.3% (*n* = 4) of cases while the aortic valve was repaired in 22.2% (*n* = 14) of cases. A Bentall procedure was performed in 36.5% of cases (*n* = 23). Hemiarch repair was performed in 87.3% (*n* = 55) of cases, defined as an open zone 0 distal anastomosis extending into the lesser curve of the arch without reconstruction of the head vessels or use of an arch device. An AMDS was used in 34.9% (*n* = 22) of cases and a TAR in 12.7% (*n* = 8) of cases. Concomitant procedures included coronary artery bypass grafting (*n* = 5, 7.9%) and mitral valve interventions (*n* = 2, 3.2%)(Table 1).

### Proximal aortic remodeling based on head vessel pathology and surgical repair

Measures of aortic remodeling are summarized in this results section and reported in full in the Supplementary Materials. Of the 63 patients included in this study, 8 underwent TAR and were excluded from the proximal measurements, leaving 55 patients with proximal aortic measurements.

**Fig. 1:**
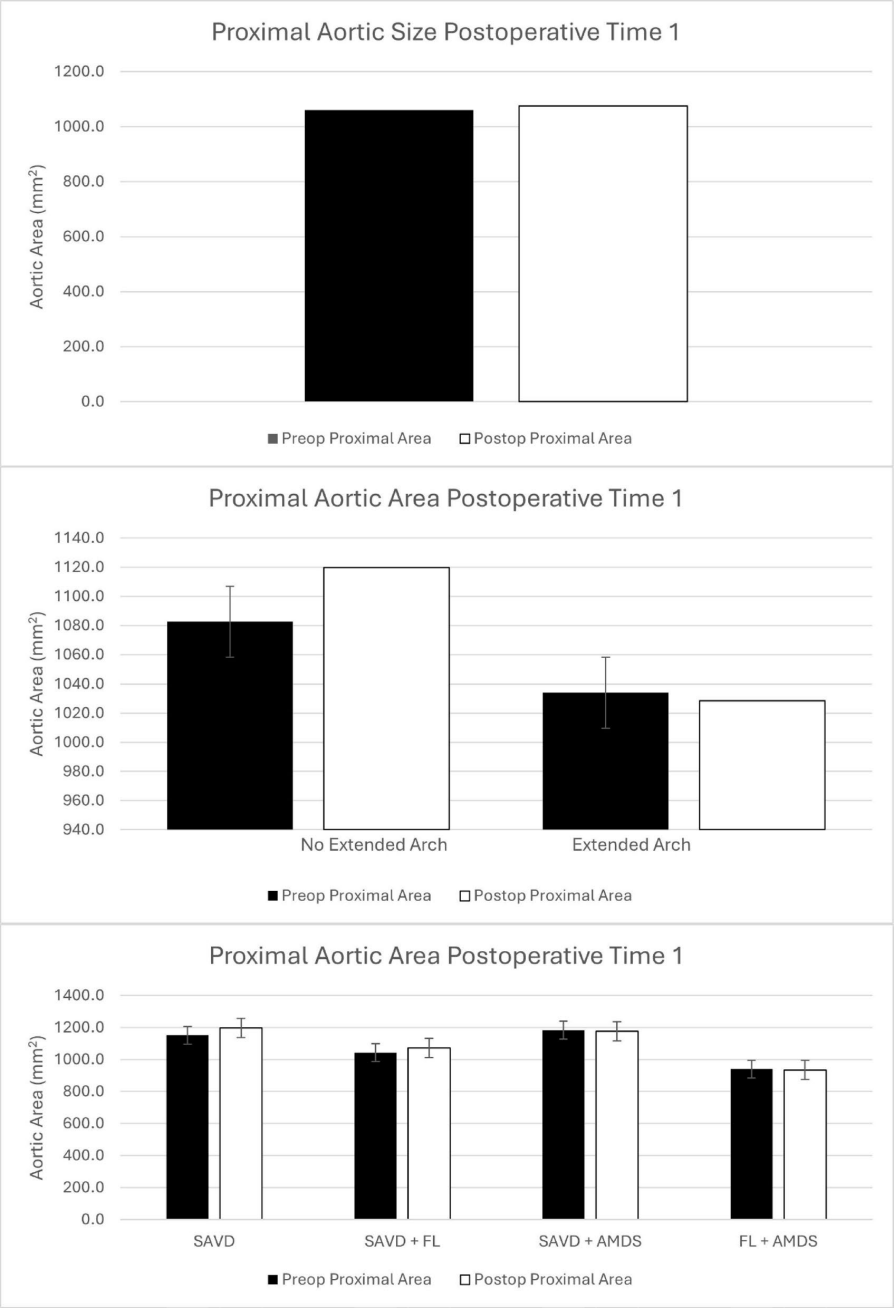
Proximal aortic remodeling at time 1 for the entire cohort (top), comparing no extended arch vs. extended arch repair (middle), and comparing patients with supraaortic vessel dissection (SAVD), SAVD + false lumen communications (FL), SAVD + AMDS, and FL + AMDS (bottom).

**Fig. 2:**
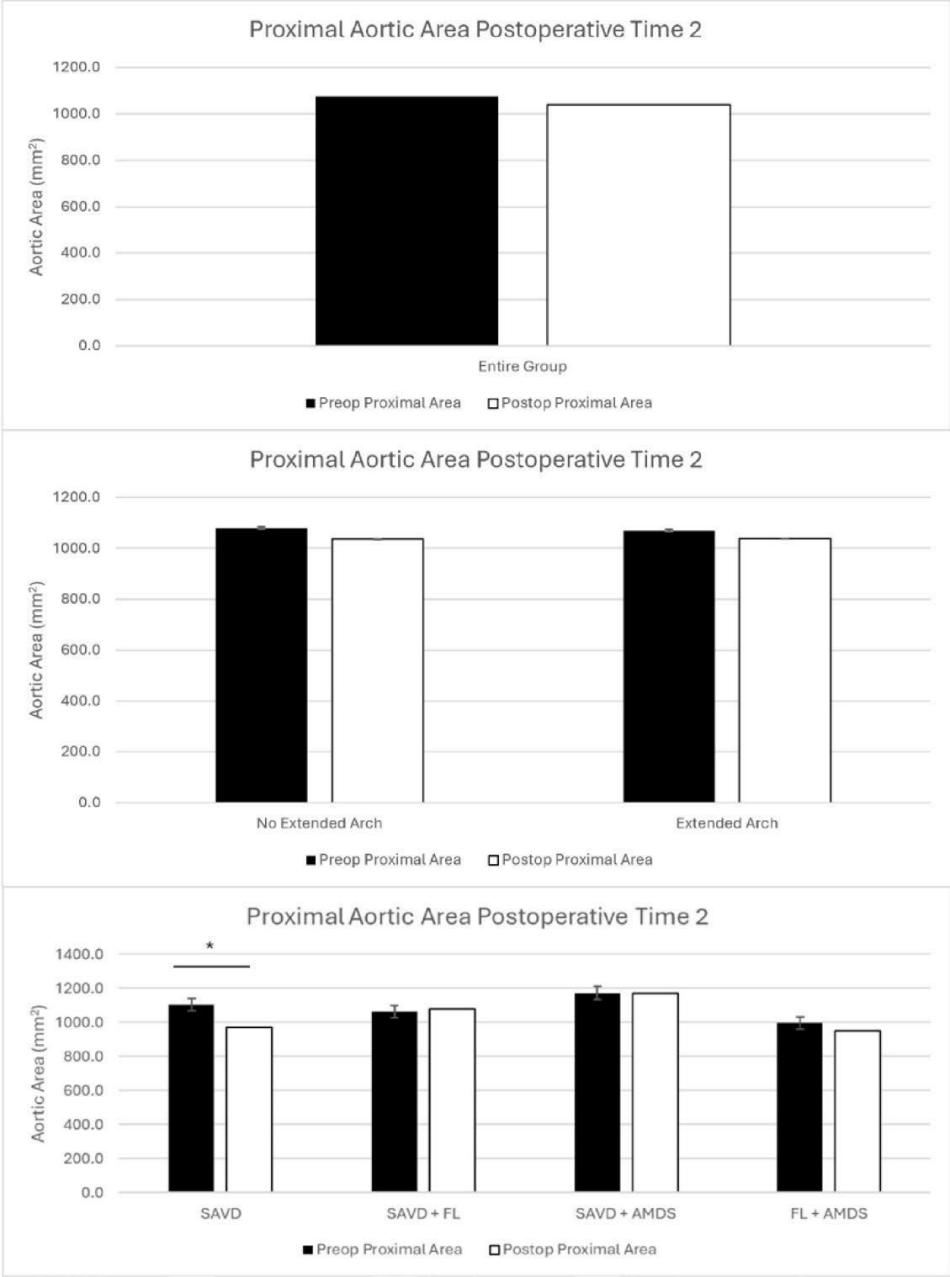
Proximal aortic remodeling at time 2 for the entire cohort (top), comparing no extended arch vs. extended arch repair (middle), and comparing patients with supraaortic vessel dissection (SAVD), SAVD + false lumen communications (FL), SAVD + AMDS, and FL + AMDS (bottom).

Average aortic area for the entire cohort did not change significantly at time 1 (5.0 months, *p* = 0.3) or time 2 (19.0 months, *p* = 0.17)(Table 2; Figs. 1 and 2).

**Table 2:** Preoperative and postoperative measurements of aortic area based on extent of arch repair.

Group	Time	*N*	Follow-up Time (months)	Preoperative Aortic Area (mm^2^)	Preoperative Maximal Aortic Diameter (mm)	Postoperative Aortic Area (mm^2^)	Postoperative Maximal Aortic Diameter (mm)	*P*-value
Proximal Entire Cohort	Time 1	48	5.0	1059.3	38.5	1076.0	38.5	0.3
(*N* = 55, Excluding TAR patients)	Time 2	55	19.0	1075.0	38.8	1037.7	37.9	0.17
Proximal No Extended Arch	Time 1	29	5.2	1082.6	38.9	1119.7	39.7	0.19
	Time 2	33	20.1	1079.1	38.6	1037.0	37.9	0.21
Proximal Extended Arch	Time 1	18	5.0	1034.0	38.5	1028.5	37.9	0.46
	Time 2	22	17.4	1068.9	39.1	1038.7	38.1	0.32
Proximal SAVD	Time 1	11	5.8	1150.3	39.7	1196.8	42.1	0.27
	Time 2	13	23.2	1105.0	39.0	970.6	36.0	**0.03**
Proximal FL	Time 1	18	4.8	1041.3	38.3	1072.6	38.2	0.27
	Time 2	20	18.2	1062.2	38.4	1080.1	39.1	0.40
Proximal SAVD + AMDS	Time 1	7	5.3	1182.7	40.7	1175.7	40.6	0.47
	Time 2	9	19.0	1172.6	41.4	1169.4	41.0	0.49
Proximal FL + AMDS	Time 1	11	4.9	939.3	36.5	934.9	35.6	0.47
	Time 2	13	16.2	997.1	37.2	948.2	36.5	0.28
Distal Entire Cohort	Time 1	53	5.2	916.2	35.4	1108.3	39.2	**< 0.001**
(*N* = 63, entire cohort)	Time 2	63	18.7	941.0	35.9	1137.4	39.0	**< 0.001**
Distal No Extended Arch	Time 1	30	5.0	928.0	36.0	1049.5	38.0	**0.01**
	Time 2	33	20.1	935.4	35.8	1111.8	38.1	**0.007**
Distal Extended Arch	Time 1	23	5.2	900.9	35.0	1185.1	41.3	**< 0.001**
	Time 2	30	17.2	947.1	36.0	1165.5	40.1	**0.008**
Distal SAVD	Time 1	12	5.4	948.5	36.0	1156.5	39.8	**0.02**
	Time 2	13	23.2	979.2	36.6	1107.2	37.4	0.16
Distal FL	Time 1	18	4.8	914.3	35.4	978.1	36.2	0.13
	Time 2	20	18.2	907.0	35.2	1114.8	38.5	**0.007**
Distal SAVD + AMDS	Time 1	7	5.3	990.5	36.5	1206.4	42.2	**0.04**
	Time 2	9	19.0	987.0	36.6	1218.2	41.8	0.09
Distal FL + AMDS	Time 1	11	4.9	852.3	34.6	1160.2	41.0	**< 0.001**
	Time 2	13	16.2	883.0	35.0	1146.1	39.3	**0.01**
TAR	Time 1	5	6.0	882.3	34.0	1210.1	40.8	**0.03**
	Time 2	8	16.6	1006.5	37.1	1137.9	39.4	0.29

When patients were broadly grouped into those who underwent hemiarch repair or extended arch repair, there was no difference between groups at either follow-up time. At time 1, patients without an extended arch repair experienced non-significant growth (5.0 months, *p* = 0.19), whereas a nonsignificant decrease in aortic area was found in the extended arch group (5.0 months, *p* = 0.46). Similarly, at time 2, patients without an extended arch repair (20.1 months, *p* = 0.21) and with an extended arch repair (17.4 months, *p* = 0.32) experienced non-significant reductions in their aortic size(Table 2; Figs. 1 and 2).

When the patients were divided based on surgical repair and FL communications, there continued to be no significant differences between groups. Patients in the SAVD group (5.8 months, *p* = 0.27), FL group (4.8 months, 0.27), with SAVD who received AMDS (5.3 months, *p* = 0.47), and for those with FL and AMDS (4.9 months, *p* = 0.47) did not experience significant growth at time 1. At time 2, aortic size in the SAVD group decreased significantly (23.2 months, *p* = 0.03), but did not change in the FL group (18.2 months, *p* = 0.40), SAVD + AMDS group (19.0 months, *p* = 0.49), or FL + AMDS group (16.2 months, *p* = 0.28)(Table 2; Figs. 1 and 2).

### Distal aortic remodeling based on head vessel pathology and surgical repair

**Fig. 3:**
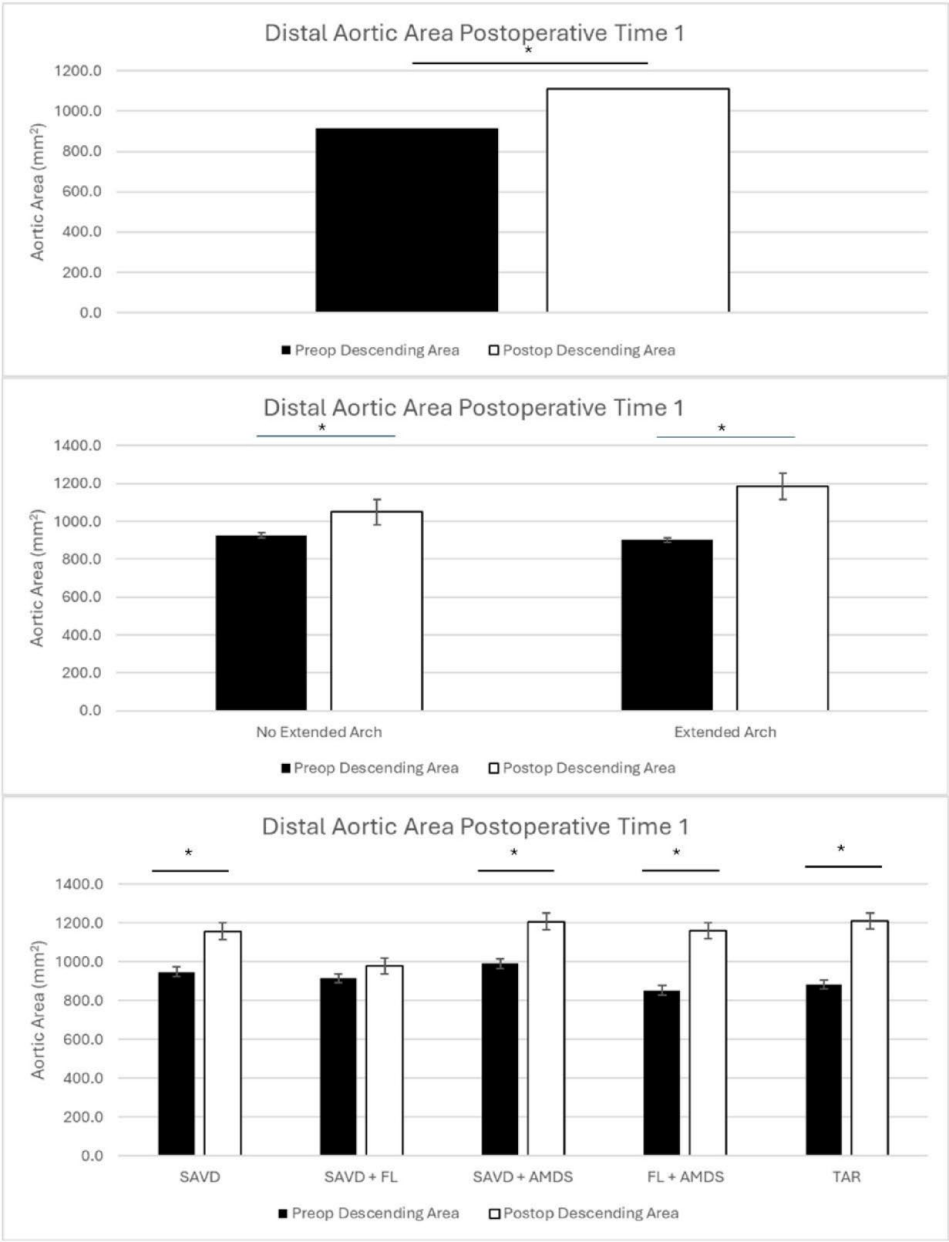
Distal aortic remodeling at time 1 for the entire cohort (top), comparing no extended arch vs. extended arch repair (middle), and comparing patients with supraaortic vessel dissection (SAVD), SAVD + false lumen communications (FL), SAVD + AMDS, FL + AMDS, and total arch repair (TAR) (bottom).

**Fig. 4:**
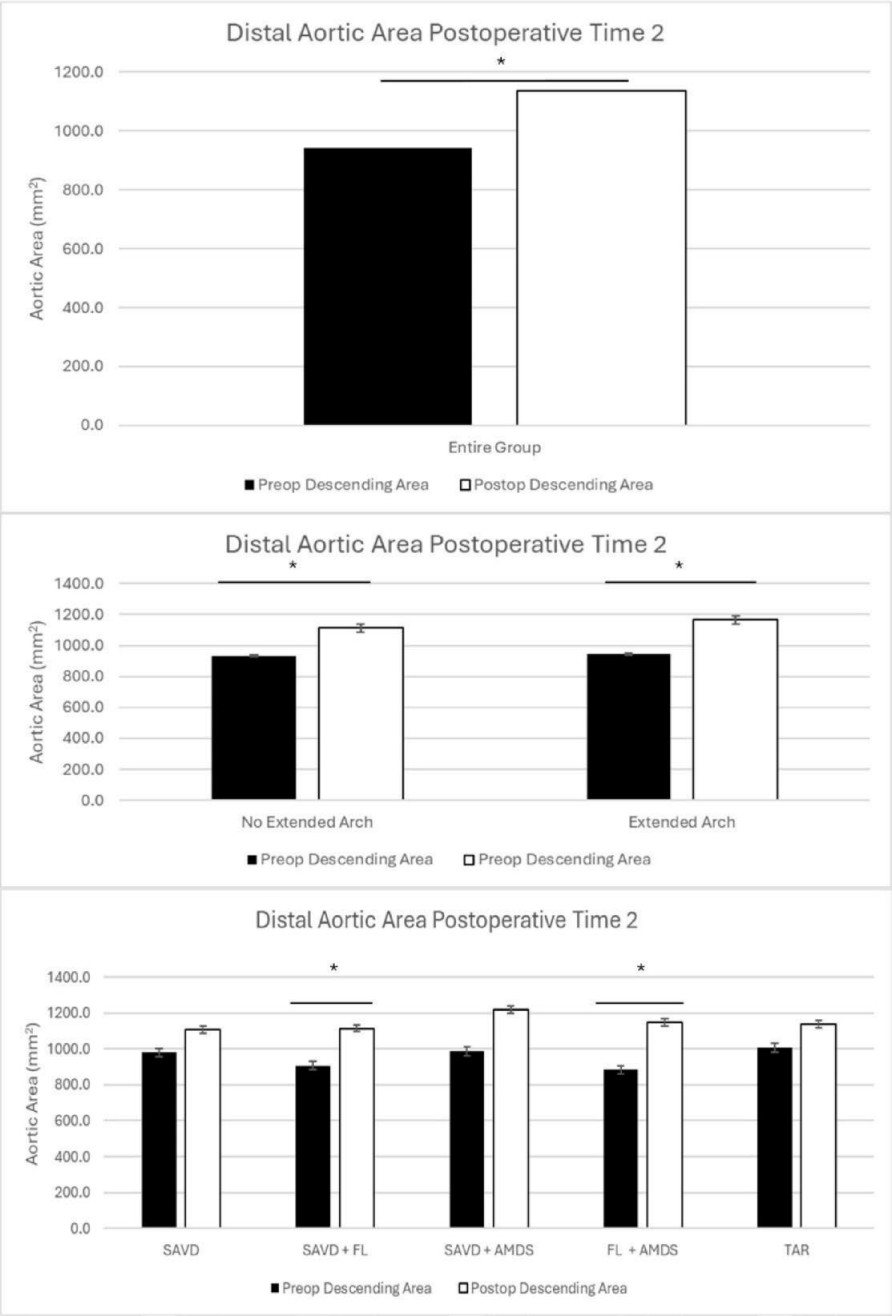
Distal aortic remodeling at time 2 for the entire cohort (top), comparing no extended arch vs. extended arch repair (middle), and comparing patients with supraaortic vessel dissection (SAVD), SAVD + false lumen communications (FL), SAVD + AMDS, FL + AMDS, and total arch repair (TAR) (bottom).

The entire cohort of patients had significant growth of the distal aorta at time 1 (5.2 months, *p* < 0.001) and time 2 (18.7 months, *p* < 0.001)(Table 2; Figs. 3 and 4).

When divided into patients who underwent hemiarch or extended arch replacement, both groups had significant growth at time 1(5.0 months, hemiarch *p* = 0.01; 5.2 months, extended arch *p* < 0.001) and time 2 (18.7 months, hemiarch *p* = 0.007; 20.1 months, extended arch *p* = 0.008))(Table 2; Figs. 3 and 4).

When examining patients based on their surgical repair and presence of FL communications, only patients with FL communications and a hemiarch repair did not have significant growth(4.8 months, *p* = 0.13). The groups of SAVD (5.4 months, *p* = 0.02), SAVD + AMDS (5.3 months, *p* = 0.04), FL + AMDS (4.9 months, *p* < 0.001), and TAR (6.0 months, *p* = 0.03) had significant growth. At time 2, this trend changed with only patients in the FL group (18.2 months, *p* = 0.007) and FL + AMDS group (16.2 months, *p* = 0.01) experiencing significant growth. Patients in the SAVD (23.2 months, *p* = 0.16), SAVD + AMDS (19.0 months, *p* = 0.09), and TAR (16.6 months, *p* = 0.29) groups did not have significant growth(Table 2; Figs. 3 and 4).

### Proximal aortic remodeling based on visceral false lumen communications

**Fig. 5:**
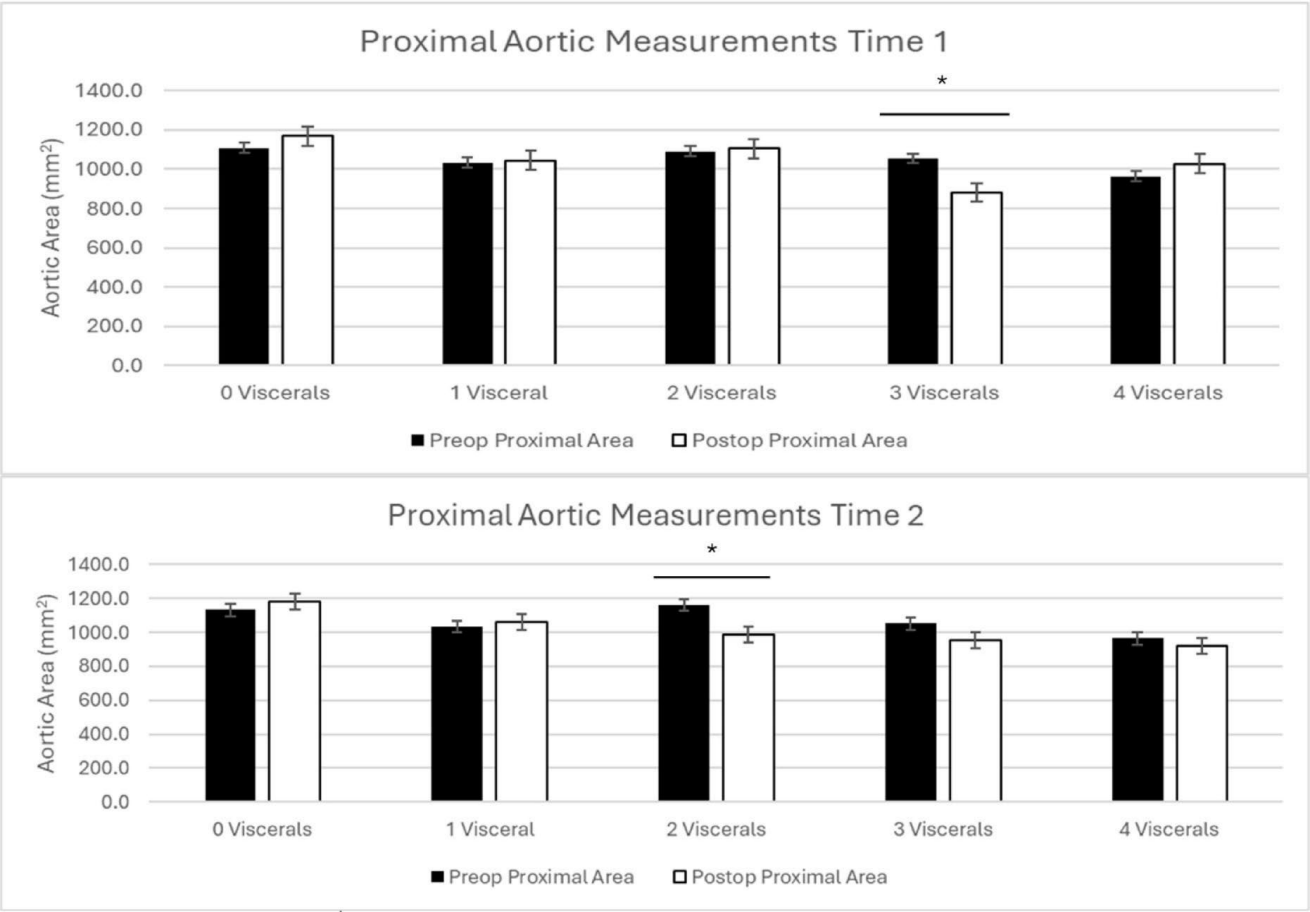
Proximal aortic remodeling at time 1 (top) and time 2 (bottom) with patients grouped based on visceral vessel involvement.

When grouped based on visceral involvement at time 1, patients with 0 (4.8 months, *p* = 0.16), 1 (5.8 months, *p* = 0.4), 2 (4.1 months, *p* = 0.4), and 4 (5.7 months, *p* = 0.3) dissected visceral vessels did not have a significant change in aortic size between zones 4/5. Patients with 3 involved visceral vessels had a significant decrease in aortic size (5.4 months, *p* = 0.02)(Table 3; Fig. 5).

**Table 3:** Preoperative and postoperative measurements of aortic area based on visceral vessel involvement.

Group	# of Involved Visceral Vessels	*N*	Follow-up Time (months)	Preoperative Measurement (mm^2^)	Postoperative Diameter (mm)	Postoperative Measurement (mm^2^)	Postoperative Diameter (mm)	*P*-value
Proximal Time 1	0	14	4.8	1108.5	39.7	1168.4	40.5	0.16
	1	10	5.8	1032.1	38.1	1044.1	37.8	0.4
	2	11	4.1	1091.1	38.6	1104.2	39.2	0.4
	3	7	5.4	1054.0	37.9	880.5	35.6	**0.02**
	4	3	5.7	963.7	36.0	1027.7	37.0	0.3
Proximal Time 2	0	13	17.4	1132.0	40.2	1179.8	40.7	0.3
	1	13	15.7	1034.7	37.6	1060.7	38.5	0.3
	2	13	26.2	1159.4	39.8	985.6	37.4	**0.01**
	3	9	16.1	1050.7	38.9	952.4	36.6	0.1
	4	3	21.7	963.7	36.0	918.7	35.7	0.4
Distal Time 1	0	15	4.9	848.9	34.6	902.9	35.1	0.06
	1	10	5.8	939.9	35.6	1096.4	39.7	**0.01**
	2	11	4.1	966.6	36.0	1127.4	40.5	**0.04**
	3	9	5.8	902.7	35.0	1204.1	41.0	**0.03**
	4	4	5.8	924.7	35.0	1412.8	44.3	**0.008**
Distal Time 2	0	17	18.7	850.6	34.7	877.8	34.2	0.3
	1	14	15.6	934.6	35.6	1111.7	38.5	0.05
	2	14	25.9	1050.4	37.9	1133.8	39.2	0.3
	3	11	16.4	946.0	35.8	1429.0	43.9	**0.003**
	4	4	19.3	924.7	35.0	1487.2	45.5	**0.008**

At time 2, patients with 0 (17.4 months, *p* = 0.3), 1 (15.7 months, *p* = 0.3), 3 (16.1 months, *p* = 0.1), and 4 (21.7 months, *p* = 0.4) dissected visceral vessels did not have a significant change in aortic size between zones 4/5. Patients with 2 involved visceral vessels had a significant decrease in aortic size (26.2 months, *p* = 0.01)(Table 3; Fig. 5).

### Distal aortic remodeling based on visceral false lumen communications

**Fig. 6:**
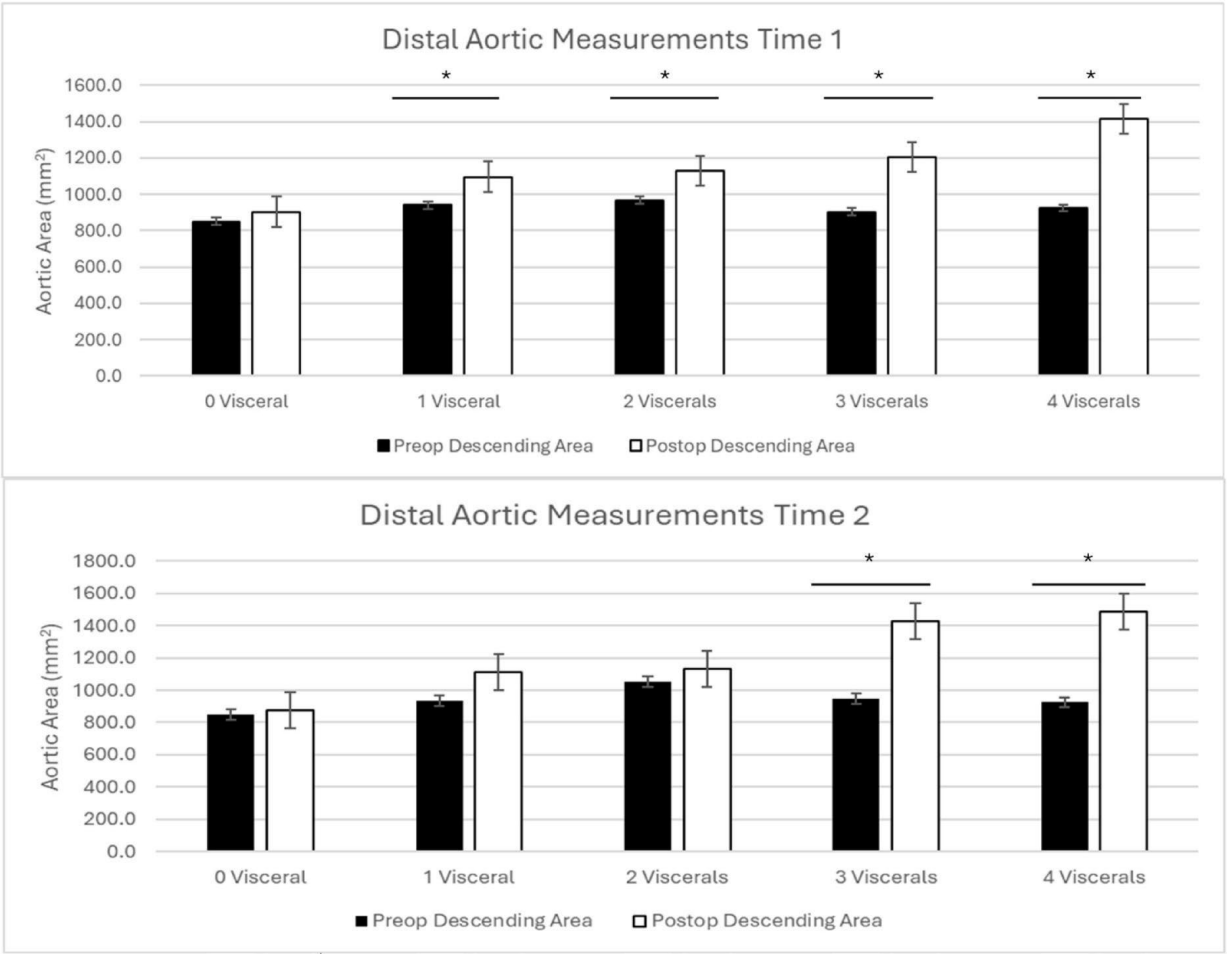
Distal aortic remodeling at time 1 (top) and time 2 (bottom) with patients grouped based on visceral vessel involvement.

For patients with 0 visceral vessel involvement, there was no significant growth of the distal aorta (*p* = 0.06). Patients with 1 (*p* = 0.01), 2 (*p* = 0.04), 3 (*p* = 0.02), and 4 (*p* = 0.008) visceral vessels all had significant growth at time 1(Table 3; Fig. 6).

At time 2, patients with 0, 1 and 2 visceral vessels did not have significant growth (*p* = 0.3, *p* = 0.05, and *p* = 0.3, respectively). Patients with 3 and 4 involved visceral vessels had significant growth of the aorta (3 viscerals *p* = 0.003, 4 viscerals *p* = 0.008)(Table 3; Fig. 6).

### Independent predictors of aortic remodeling

In the multivariate analysis, neither head vessel FL communications, repair type, or visceral vessel involvement were independent predictors of aortic remodeling for the proximal measurements at time 1 or time 2 (Table 3). At the distal measurement, head vessel FL communications and repair type also did not predict aortic change.

At the distal measurements at time 1 and time 2, the number of visceral vessels involved, being ≥ 3, independently predicted aortic growth. At time 1 compared to the reference group of 0 involved visceral vessels, involvement of 1 visceral vessel was associated with 96mm^2^ of growth (*p* = 0.477), involvement of 2 visceral vessels was associated with 28mm^2^ of growth (*p* = 0.823), and involvement of ≥ 3 visceral vessels was associated with 238mm^2^ of aortic growth (*p* = 0.04). At time 2 compared to the reference group of 0 involved visceral vessels, involvement of 1 visceral vessel was associated with 52mm^2^ of growth (*p* = 0.765), involvement of 2 visceral vessels was associated with − 76mm^2^ of growth (*p* = 0.661), and involvement of ≥ 3 visceral vessels was associated with 461mm^2^ of aortic growth (*p* = 0.005)(Table 4).

**Table 4:** Linear regression for independent predictors of adverse aortic remodeling.

Predictors	Coefficient (Change in aortic area (mm^2^))	*P* value
**PROXIMAL TIME 1 (*N*** **= 48)**		
**Groups**		
SAVD	Reference	Reference
FL	60	0.549
FL + AMDS	20	0.859
SAVD + AMDS	-85	0.464
TAR	-105	0.376
**# of Involved Visceral Vessels**		
0	Reference	Reference
1	75	0.500
2	8	0.939
3+	-151	0.107
**PROXIMAL TIME 2 (*N*** **= 55)**		
**Groups**		
SAVD	Reference	Reference
FL	179	0.091
FL + AMDS	116	0.341
SAVD + AMDS	143	0.248
TAR	-75	0.532
**# of Involved Visceral Vessels**		
0	Reference	Reference
1	-96	0.375
2	-205	0.061
3+	-96	0.331
**DISTAL TIME 1 (*N*** **= 53)**		
**Groups**		
SAVD	Reference	Reference
FL	234	0.061
FL + AMDS	98	0.479
SAVD + AMDS	-6	0.965
TAR	18	0.900
**# of Involved Visceral Vessels**		
0	Reference	Reference
1	96	0.477
2	28	0.823
3+	238	**0.040**
**DISTAL TIME 2 (*N*** **= 63)**		
**Groups**		
SAVD	Reference	Reference
FL	16	0.925
FL + AMDS	185	0.342
SAVD + AMDS	-1	0.996
TAR	-201	0.296
**# of Involved Visceral Vessels**		
0	Reference	Reference
1	52	0.765
2	-76	0.661
3+	461	**0.005**

Predictors in all 4 models: pathology groups, numbers of involved visceral vessels, age, sex, hypertension, dyslipidemia, current smoker, chronic obstructive pulmonary disease, diabetes, congestive heart failure, coronary artery disease, atrial fibrillation, cerebrovascular disease, aortic rupture, and tamponade.

Abbreviations: SAVD, supraaortic vessel dissection; FL, false lumen; TAR, total arch repair.

### Secondary outcomes

The mean intensive care unit length of stay was 9.5 days while the mean hospital length of stay was 22.2 days. Mortality between 1 and 5 years was 3.2% (*n* = 2), as the inclusion criteria required survival to 1 year no patients that passed away prior to 1 year CT scan were included. Postoperative complications included atrial fibrillation (*n* = 20, 31.7%), cerebrovascular accident (*n* = 17, 27%), reoperation (*n* = 13, 20.6%), major bleeding (*n* = 6, 9.5%), sepsis (*n* = 4, 6.3%), and spinal cord injury (*n* = 1, 1.6%)(Table 5).

**Table 5:** Postoperative outcomes of patients who underwent repair of acute type A aortic dissection.

Postoperative Outcomes	*N* = 63 (%)
Mean ICU LOS (Days)	9.5
Mean Hospital LOS (Days)	22.2
Operative Mortality	0
1 Year Mortality	0
5 Year Mortality	2 (3.2)
Postoperative Atrial Fibrillation	20 (31.7)
Cerebrovascular Accident	17 (27.0)
Reoperation	13 (20.6)
Major Bleeding	6 (9.5)
Sepsis	4 (6.3)
Spinal Cord Injury	1 (1.6)

ICU, intensive care unit; LOS, length of stay.

## Discussion

In this study, we investigated the relationship between branch vessel involvement and surgical repair in ATAAD with postoperative aortic remodeling. There were several notable findings in this study. First, proximal aortic remodeling was not predicted by head vessel FL communications or surgical repairwhile distal aortic size was significantly increased for the entire cohort, for groups with or without extended arch repairs, and with a potential relationship with head vessel FL communications after 1 year. At time 1, patients with 1 or more dissected visceral vessels had significant growth of their aorta. At time 2, only patients with 3 or 4 dissected visceral vessels had significant growth of their distal aorta. This was confirmed in a multivariate analysis which found only the involvement of ≥ 3 visceral vessels to be predictive of distal aortic growth.

Previous investigations into this field have largely been limited to type B dissections and have sought to elucidate risk factors for aortic growth such as the size of entry tears, FL thrombosis, and FL size^9–11^. For example, Spinelli et al. demonstrated an association between absolute FL size and FL size relative to the TL size predicted adverse outcomes^11^. They also found that increased numbers of FL communications were protective against FL expansion, likely due to the decompression effect of the re-entry tears. Previous MRI studies have demonstrated changes in flow related to visceral branch vessels arising from the FL^10^. Burris et al. performed magnetic resonance imaging (MRI) in order to quantify FL ejection fraction in patients with type B aortic dissection. They found that in addition to maximal baseline diameter and entry tear distance from the subclavian artery, FL ejection fraction was predictive of aortic growth rate^12^. Finally, Evangelista and colleagues conducted a study of 131 patients with type A or B dissection where MRI was used to analyze FL flows. Increased antegrade systolic FL flow along with retrograde diastolic flow were predictors of aortic events and aortic enlargement^13^.

Our findings of a relationship between distal aortic remodeling and visceral vessel involvement may be explained by several factors. First, FL growth requires continuous FL perfusion and pressurization. FL communications in the head vessels are not addressed by a standard hemiarch repair or bare metal stent. The presence of patent FL communications may result in FL pressurization and continued aortic degeneration. This relationship is clearly illustrated in our results with patients with 3 or 4 dissected visceral vessels experiencing adverse remodeling past 1 year. This relationship was not clearly demonstrated proximally in the aortic arch. While the principles remain the same, differing interventions may address these communications preventing adverse remodeling. As cardiac surgeons, there may be more attention paid to aortic arch anatomy at the time of ATAAD allowing for the identification of high-risk features in the aorta and either modification of the surgical plan to include an arch intervention or proceeding with hemiarch repair and referring the patient for follow-up with an aortic surgeon or clinic where their aortic arch may be monitored and intervened upon at a later time. The data presented in this study may help to identify high-risk features in the distal aorta and may help to identify patients who require additional intervention after their initial ATAAD repair. Patients who are found to have visceral vessel involvement in ATAAD may benefit from close monitoring with serial imaging in order to identify progression of abdominal aortic disease.

As among the first studies to identify a relationship between visceral vessel dissection and adverse aortic remodeling post ATAAD repair, there are key points that should be taken away. First, continuing to identify high-risk aortic arches, such as aortic arch tears or head vessel involvement, and either addressing them at the time of initial ATAAD repair or with a subsequent referral to an aortic surgeon for a staged repair is imperative to prevent adverse remodeling of the arch. Second, the abdominal aorta and visceral branches should be considered in future ATAAD cases where the addition of a FET, TEVAR, or referral for staged repair may help to prevent distal aortic dilation. Greater focus on the visceral vessels, both in research and clinical settings, such as identification of visceral vessel involvement and determination of the impact of FL communications in the visceral vessels will be imperative in understanding the long term course of these patients. This area of study will require further investigation utilizing larger sample sizes and ideally 3-dimensional reconstruction of the aorta to facilitate the collection of more detailed measurements across several segments of the aorta. The follow-up in this study was limited and longer follow-up will be necessary to determine the long-term course of aortic remodeling and the resulting required reinterventions in these patients. Finally, quantification of flow across FL communications using MRI will help to better define high-risk branch vessel dissections by determining whether larger FL communications or even larger dissected branch vessels contribute to increased FL flow and subsequent dilation.

### Limitations

This study has several limitations. First, the data collected was retrospective and from a single center, limiting the generalizability of the data and our ability to randomize patients to treatment. Second, while the sample size is relatively small, statistical significance was achieved in several comparisons including a multivariate analysis suggesting a large effect size, although larger sample sizes will be required to confirm these findings and may unveil additional trends in future studies. Finally, certain imaging details may be beneficial in future studies to further improve our understanding of aortic remodeling including 3-dimensional reconstructions of the aorta, correlation with distal re-entry tears, and measurements at multiple sites.

## Conclusions

The results of this study have identified an association between visceral vessel dissection and distal aortic remodeling. Given this association, greater attention should be paid to the visceral vessels in ATAAD. While visceral vessel dissection does not warrant descending aortic intervention at the time of emergent ATAAD repair, these patients should be closely followed to identify distal aortic aneurysmal degeneration early, allowing for early intervention.

## Methods

### Patients

We performed a retrospective study of consecutive patients who underwent ATAAD repair from January 2017 to March 2023. The inclusion criteria were patients who had experiend ATAAD, and underwent surgical repair. Patients were required to have DeBakey I aortic dissection and with involvement of at least one head vessel preoperatively (supraaortic vessel dissection, SAVD) with or without any FL communications at any location in the main body of the head vessels. Patients were also required to have a postoperative computerized tomography(CT) scan at least 1 year post-ATAAD repair in order to assess remodeling. There was no requirement of false lumen patency post-repair. False lumen communications were defined as a tear between the true and false lumens in any of the head vessel branches.

While additional imaging data prior to 1 year was included in this study, it was not mandatory for inclusion which resulted in not all patients having a Time 1 CT scan. Exclusion criteria included patients < 18 years of age, patients without adequate preoperative and postoperative CT scans to measure aortic size, and those without at least 1 year follow-up imaging. Patients who did not survive to 1 year of follow-up were excluded from this study, as this was a study of aortic remodeling, not survival. The local University of Alberta ethics review board approved this study on March 8, 2022 for study ID #Pro00124716 with individual waiver of informed consent. All methods were performed in accordance with the relevant guidelines and regulations, including local protocols. This study was conducted in accordance with the Declaration of Helsinki.

### Aortic measurements

Aortic measurements were collected from preoperative CT scans and postoperative CT scans within 1 year(time 1) and after 1 year(time 2) postoperatively. Proximal aortic measurements were taken in zone 1 and distal aortic measurements were taken in the descending thoracic aorta at the level of the tracheal bifurcation, between zones 4 and 5. Proximal aortic measurements were not collected for patients who received TAR as this part of the aorta would have been resected. Measurements were taken manually using centerline by one author, measuring the diameter of the aorta from edge to edge in the sagittal and coronal planes. The two measurements were then taken and inputted into a formula Aortic area (mm^2^) = π x radius 1(mm) x radius 2(mm) to obtain a measurement of aortic area at those sites.

CT scans were also inspected for involvement of the head vessels and visceral vessels. Branch vessel dissections were defined as any dissection involving the head vessels (innominate, left common carotid, and left subclavian), celiac, superior mesenteric, and left or right renal arteries. FL communications were defined as a tear or communication between the TL and FL in any of the head vessels. Patients with head vessel dissections were grouped as SAVD, while patients with FL communications were grouped as FL.

### Outcomes

The primary outcome was aortic remodeling at postoperative times 1 and 2. Positive aortic remodeling included reductions in overall aortic size while negative aortic remodeling is defined as increases in aortic size. Secondary outcomes included rates of mortality at 1 and 5 years, cerebrovascular accident, bleeding, reoperation, postoperative atrial fibrillation, sepsis, and spinal cord injury.

### Statistical analysis

All patients had a postoperative CT scan at time 2, while some patients did not have a CT scan at time 1. Any patients without a CT scan at time 1 were excluded from the time 1 comparisons with both preoperative and postoperative measurements excluded from the time 1 analyses. Any patients without a time 2 CT scan were excluded from the study entirely. Groups included the entire cohort which was then divided into those who underwent extended arch repairs (TAR, AMDS Hybrid Prosthesis(Artivion, GA, USA)) or hemiarch repair. Patients were then further divided into whether or not they had a head vessel FL communication and into specific surgical repairs. Finally, patients were grouped based on the number of dissected visceral vessels. Each of these groups were compared between their mean preoperative and postoperative aortic area for proximal and distal measurements. Paired t-tests were used to compare aortic growth between preoperative and postoperative measurements for each group.

Multivariable linear regression models were performed to examine if pathology, surgical intervention, or visceral vessel involvement are independent predictors of aortic remodeling after adjusting for baseline characteristics. The predicters included in the models were pathology, numbers of visceral vessel involved, age, sex, hypertension, dyslipidemia, current smoker, chronic obstructive pulmonary disease, diabetes, congestive heart failure, coronary artery disease, atrial fibrillation, cerebrovascular disease, aortic rupture, tamponade. Four outcomes, including proximal postoperative aortic area changes at time 1(mm^2^), proximal postoperative aortic area changes at time 2(mm^2^), distal postopertive aortic area changes at time 1(mm^2^), and distal postoperative aortic area changes at time 2(mm^2^), were examined in separate models. Statistical analyses were executed using the SAS 9.4.(SAS Institute, Cary NC) A p-value < 0.05 was deemed statistical significance. All statistical tests were two-sided.

## Electronic supplementary material

Below is the link to the electronic supplementary material.

Supplementary Material 1

## Data Availability

The raw data associated with the article is not permitted to be shared externally. The corresponding author, Michael Moon, should be contacted for any queries related to the data in main manuscript file and on system.

## References

[1] Isselbacher, E. M. et al. Peer review committee members. 2022 ACC/AHA guideline for the diagnosis and management of aortic disease: A report of the American heart association/american college of cardiology joint committee on clinical practice guidelines. *Circulation* **146** (24), e334–e482 (2022). 36322642 10.1161/CIR.0000000000001106 PMC9876736

[2] Bozso, S. J., White, A., Nagendran, J., Moon, M. C. & Chu, M. W. A. Hybrid aortic arch and frozen elephant trunk reconstruction: bridging the gap between conventional and total endovascular arch repair. *Expert Rev. Cardiovasc. Ther.* **16** (3), 209–217 (2018). 29343137 10.1080/14779072.2018.1429913

[3] Assi, R., Vallabhajosyula, P. & Szeto, W. Y. Hybrid techniques for surgical repair of acute type A aortic dissection. *Endovas Today*. **17** (11), 77–80 (2018).

[4] White, A., Bozso, S. J., Ouzounian, M., Chu, M. W. A. & Moon, M. C. Canadian thoracic aortic collaborative. Acute type A aortic dissection and the consequences of a patent false lumen. *JTCVS Tech.* **9** (C), 1–8 (2021). 34647041 10.1016/j.xjtc.2021.05.002 PMC8500985

[5] Bozso, S. J. et al. Dissected aorta repair through stent implantation trial: Canadian results. *J. Thorac. Cardiovasc. Surg.* **157** (5), 1763–1771 (2019). 30501947 10.1016/j.jtcvs.2018.09.120

[6] Bozso, S. J. et al. Three-year outcomes of the dissected aorta repair through stent implantation trial. *J. Thorac. Cardiovasc. Surg.* **167** (5), 1661–1669 (2022). 36220703 10.1016/j.jtcvs.2022.08.040

[7] Bozso, S. J. et al. Midterm outcomes of the dissected aorta repair through stent implantation trial. *Ann. Thorac. Surg.* **111** (2), 463–470 (2021). 32673661 10.1016/j.athoracsur.2020.05.090

[8] Brown, J. A., Arnaoutakis, G. J., Szeto, W. Y., Serna-Gallegos, D. & Sultan, I. Endovascular repair of the aortic arch: state of the Art. *J. Card Surg.* **36** (11), 4292–4300 (2021). 34405439 10.1111/jocs.15920

[9] Lee, J. H. et al. Changes in aortic growth rate and factors influencing aneurysmal dilatation after uncomplicated acute type B aortic dissection. *Interact. Cardiovasc. Thorac. Surg.* **35** (3), 1–8 (2022). 10.1093/icvts/ivac126 PMC9419697 35512382

[10] Rudenick, P. A. et al. False lumen flow patterns and their relation with morphological and Biomechanical characteristics of chronic aortic dissections. Computational model compared with magnetic resonance imaging measurements. *PLoS One*. **12** (1), 1–20 (2017). 10.1371/journal.pone.0170888 PMC5270334 28125720

[11] Spinelli, D. et al. Current evidence in predictors of aortic growth and events in acute type B aortic dissection. *J. Vasc Surg.* **68** (6), 1925–1935e8 (2018). 30115384 10.1016/j.jvs.2018.05.232

[12] Burris, N. S. et al. False lumen ejection fraction predicts growth in type B aortic dissection: preliminary results. *Eur. J. Cardio-Thorac Surg.* **57** (5), 896–903 (2020). 10.1093/ejcts/ezz343 PMC8453376 31821480

[13] Evangelista, A. et al. False lumen flow assessment by magnetic resonance imaging and Long-Term outcomes in uncomplicated aortic dissection. *J. Am. Coll. Cardiol.* **79** (24), 2415–2427 (2022). 35710193 10.1016/j.jacc.2022.04.017

