# Correction: Anatomic and operative predictors of aortic expansion following aortic dissection repair

**DOI:** 10.1038/s41598-025-14932-x

**Published:** 2025-08-14

**Authors:** Ryaan EL-Andari, Sabin Bozso, Yongzhe Hong, Michael Moon

**Affiliations:** 1 Division of Cardiac Surgery, University of Alberta, Edmonton, AB Canada; 2 Mazankowski Alberta Heart Institute, University of Alberta, 11220 83 Ave NW, Edmonton, AB Canada

Correction to: *Scientific Reports* 10.1038/s41598-025-11286-2, published online 11 July 2025

The original version of this Article contained an error in the spelling of the author Michael Moon which was incorrectly given as Moon. Additionally, Michael Moon was omitted as a corresponding author. Correspondence and requests for materials should also be addressed to: mmoon@ualberta.ca.

The original Article has been corrected.

