# Supplementary material for: Anatomic and operative predictors of aortic expansion following aortic dissection repair
Source: Sci Rep. 2025 Jul 11;15:25047. doi: 10.1038/s41598-025-11286-2 (PMC12254228; doi:10.1038/s41598-025-11286-2)
Supplement: Supplementary file 1 — Supplementary Material 1 [file 41598_2025_11286_MOESM1_ESM.docx]

**Anatomic and Operative Predictors of Aortic Expansion Following Aortic Dissection Repair**

**Supplementary Material**

Ryaan EL-Andari^1^, MD, Sabin J. Bozso^1^, MD, PhD, Yongzhe Hong^1^, MD, PhD, Michael C. Moon^1^, MD.

^1^Division of Cardiac Surgery, University of Alberta, Edmonton, Alberta, Canada.

*Running Title:* Aortic Remodeling in Dissection



Disclosures: None

Funding: None

Institutional Review Board Approval: March 8, 2022 for study ID #Pro00124716

Meeting: AATS Aortic 2024

Word count: 1,198

**Corresponding Author:**

Ryaan EL-Andari, MD

University of Alberta

p:(780)248-5687

Email:elandari@ualberta.ca

**Post-publication Corresponding Author:**

Michael C. Moon, MD

Cardiac Surgeon

Director, Aortic Surgery

Director, Fellowship Training Program

Mazankowski Alberta Heart Institute

11220 83 Ave NW

University of Alberta

Edmonton, Alberta, Canada

p:780-407-6861

Email:mmoon@ualberta.ca

**Results**

*Proximal Aortic Remodeling Based on Head Vessel Pathology and Surgical Repair*

Average aortic area for the entire cohort did not change significantly at time 1 (n=48, average follow-up time 5.0 months, preoperative area 1059.3=mm^2^, postoperative area=1076.0mm^2^, p=0.3) or time 2 (n=55, average follow-up time 19.0 months, preoperative area =1075.0mm^2^, postoperative area=1037.7mm^2^, p=0.17)(Table 3, Figures 1-2).

When patients were broadly grouped into those who underwent hemiarch repair or extended arch repair, there was no difference between groups at either follow-up time. At time 1, patients without an extended arch repair experienced non-significant growth (n=29, average follow-up time 5.2 months, preoperative area = 1082.6mm^2^, postoperative area = 1119.7mm^2^, p=0.19) whereas a nonsignificant decrease in aortic area was found in the extended arch group (n=18, average follow-up time 5 months, preoperative area = 1034.0mm^2^, postoperative area = 1028.5mm^2^, p=0.46). Similarly, at time 2, patients without an extended arch repair (n=33, average follow-up time 20.1 months, preoperative area = 1079.1mm^2^, postoperative area = 1037.0mm^2^, p=0.21) and with an extended arch repair (n=22, average follow-up time 17.4 months, preoperative area = 1068.9mm^2^, postoperative area = 1038.7mm^2^, p=0.32) experienced non-significant reductions in their aortic size (Table 3, Figures 1-2).

When the patients were divided based on surgical repair and FL communications, there continued to be no significant differences between groups. Patients in the SAVD group grew from 1150.3mm^2^ to 1196.8mm^2^ (n=11, average follow-up time 5.8 months, p=0.27). Patients in the FL group grew from 1041.3mm^2^ to 1072.6mm^2^ (n=18, average follow-up time 4.8 months, p=0.27). Patients with SAVD who received AMDS experienced a change in aortic area from 1182.7mm^2^ to 1175.7mm^2^ (n=7, average follow-up time 5.3 months, p=0.47). For those with FL and AMDS aortic area changes from 939.3mm^2^ to 934.9mm^2^ (n=11, average follow-up time 4.9 months, p=0.47). At time 2, aortic size in the SAVD group changed from 1105.0mm^2^ to 970.6mm^2^ (n=13, average follow-up time 23.2 months, p=0.03), 1062.2mm^2^ to 1080.1mm^2^ (n=20, average follow-up time 18.2 months, p=0.40) in the FL group, 1172.6mm^2^ to 1169.4mm^2^ (n=9, average follow-up time 19.0 months, p=0.49) in the SAVD + AMDS group, and 997.1mm^2^ to 948.2mm^2^ (n=13, average follow-up time 16.2 months, p=0.28) in the FL + AMDS group(Table 3, Figures 1-2).

*Distal Aortic Remodeling Based on Head Vessel Pathology and Surgical Repair*

The entire cohort of patients had significant growth of the distal aorta at time 1 (n=53, average follow-up time 5.2 months, preoperative area=916.2mm^2^, postoperative area=1108.3mm^2^, p<0.001) and time 2 (n=63, average follow-up time 18.7 months, preoperative area=941.0mm^2^, postoperative area=1137.4mm^2^, p<0.001)(Table 3, Figures 3-4).

When divided into patients who underwent hemiarch or extended arch replacement, both groups had significant growth at time 1 (hemiarch n=30, average follow-up time 5.0 months, preoperative area=928.0mm^2^, postoperative area=1049.5mm^2^, p=0.01; extended arch n=23, average follow-up time 5.2 months, preoperative area=900.9mm^2^, postoperative area=1185.1mm^2^, p<0.001) and time 2 (hemiarch n=33, average follow-up time 20.1 months, preoperative area=935.4mm^2^, postoperative area=1111.8mm^2^, p=0.007; extended arch n=30, average follow-up time 17.2 months, preoperative area=947.1mm^2^, postoperative area=1165.5mm^2^, p=0.008)(Table 3, Figures 3-4).

When examining patients based on their surgical repair and presence of FL communications, only patients with FL communications and a hemiarch repair did not have significant growth (n=18, average follow-up time 4.8 months, preoperative area=914.3mm^2^, postoperative area=978.1mm^2^, p=0.13). The groups of SAVD (n=12, average follow-up time 5.4 months, preoperative area=948.5mm^2^, postoperative area=1156.5mm^2^, p=0.02), SAVD + AMDS (n=7, average follow-up time 5.3 months, preoperative area=990.5mm^2^, postoperative area=1206.4mm^2^, p=0.04), FL + AMDS (n=11, average follow-up time 4.9 months, preoperative area=852.3mm^2^, postoperative area=1160.2mm^2^, p<0.001), and TAR (n=5, average follow-up time 6.0 months, preoperative area=882.3mm^2^, postoperative area=1210.1mm^2^, p=0.03) had significant growth. At time 2, this trend changed with only patients in the FL group (n=20, average follow-up time 18.2 months, preoperative area=907.0mm^2^, postoperative area=1114.8mm^2^, p=0.007) and FL + AMDS group (n=13, average follow-up time 16.2 months, preoperative area=883.0mm^2^, postoperative area=1146.1mm^2^, p=0.01) experiencing significant growth. Patients in the SAVD (n=13, average follow-up time 23.2 months, preoperative area=979.2mm^2^, postoperative area=1107.2mm^2^, p=0.16), SAVD + AMDS (n=9, average follow-up time 19.0 months, preoperative area=987.0mm^2^, postoperative area=1218.2mm^2^, p=0.09), and TAR (n=8, average follow-up time 16.6 months, preoperative area=1006.5mm^2^, postoperative area=1137.9mm^2^, p=0.29) groups did not have significant growth(Table 3, Figures 3-4).

*Proximal Aortic Remodeling Based on Visceral False Lumen Communications*

When grouped based on visceral involvement at time 1, patients with 0 (n=14, average follow-up time 4.8 months, preoperative area=1108.5mm^2^, postoperative area=1168.4mm^2^, p=0.16), 1 (n=10, average follow-up time 5.8 months, preoperative area=1032.1mm^2^, postoperative area=1044.1mm^2^, p=0.4), 2 (n=11, average follow-up time 4.1 months, preoperative area=1091.1mm^2^, postoperative area=1104.2mm^2^, p=0.4), and 4 (n=3, average follow-up time 5.7 months, preoperative area=963.7mm^2^, postoperative area=1027.7mm^2^, p=0.3) dissected visceral vessels did not have a significant change in aortic size between zones 4/5. Patients with 3 involved visceral vessels had a significant decrease in aortic size (n=7, average follow-up time 5.4 months, preoperative area=1054.0mm^2^, postoperative area=880.5mm^2^, p=0.02)(Table 4, Figure 5).

At time 2, patients with 0 (n=13, average follow-up time 17.4 months, preoperative area=1132.0mm^2^, postoperative area=1179.8mm^2^, p=0.3), 1 (n=13, average follow-up time 15.7 months, preoperative area=1034.7mm^2^, postoperative area=1060.7mm^2^, p=0.3), 3 (n=9, average follow-up time 16.1 months, preoperative area=1050.7mm^2^, postoperative area=952.4mm^2^, p=0.1), and 4 (n=3, average follow-up time 21.7 months, preoperative area=963.7mm^2^, postoperative area=918.7mm^2^, p=0.4) dissected visceral vessels did not have a significant change in aortic size between zones 4/5. Patients with 2 involved visceral vessels had a significant decrease in aortic size (n=13, average follow-up time 26.2 months, preoperative area=1159.4mm^2^, postoperative area=985.6mm^2^, p=0.01)(Table 4, Figure 5).

*Distal Aortic Remodeling Based on Visceral False Lumen Communications*

For patients with 0 visceral vessel involvement, there was no significant growth of the distal aorta (n=15, average follow-up time 4.9 months, preoperative area = 848.9mm^2^, postoperative area=902.9mm^2^, p=0.06). Patients with 1 (n=10, average follow-up time 5.8 months, preoperative area=939.9mm^2^, postoperative area=1096.4mm^2^, p=0.01), 2 (n=11, average follow-up time 4.1 months, preoperative area=966.6mm^2^, postoperative area=1127.4mm^2^, p=0.04), 3 (n=9, average follow-up time 5.8 months, preoperative area=902.7mm^2^, postoperative area=1204.1mm^2^, p=0.02), and 4 (n=4, average follow-up time 5.8 months, preoperative area=924.7mm^2^, postoperative area=1412.8mm^2^, p=0.008) visceral vessels all had significant growth at time 1(Table 4, Figure 6).

At time 2, patients with 0, 1 and 2 visceral vessels did not have significant growth (0 viscerals n=17, average follow-up time 18.7 months, preoperative area=850.6mm^2^, postoperative area=877.8mm^2^, p=0.3, 1 visceral n=14, average follow-up time 15.6 months, preoperative area=934.6mm^2^, postoperative area=1111.7mm^2^, p=0.05, 2 viscerals n=14, average follow-up time 25.9 months, preoperative area=1050.4mm^2^, postoperative area=1133.8mm^2^, p=0.3). Patients with 3 and 4 involved visceral vessels had significant growth of the aorta (3 viscerals n=11, average follow-up time 16.4 months, preoperative area=946.0mm^2^, postoperative area=1429.0mm^2^, p=0.003, 4 viscerals n=4, average follow-up time 19.3 months, preoperative area=924.7mm^2^, postoperative area=1487.2mm^2^, p=0.008)(Table 4, Figure 6).
